# Evaluation of pre-symptomatic nitisinone treatment on long-term outcomes in Tyrosinemia type 1 patients: a systematic review

**DOI:** 10.1186/s13023-017-0696-z

**Published:** 2017-09-11

**Authors:** Julia Geppert, Chris Stinton, Karoline Freeman, Hannah Fraser, Aileen Clarke, Samantha Johnson, Paul Sutcliffe, Sian Taylor-Phillips

**Affiliations:** 1 Warwick Medical School, University of Warwick, Coventry, CV4 7AL UK; 2 Warwick Library, University of Warwick, Coventry, CV4 7AL UK

**Keywords:** Systematic review, Tyrosinemia, Nitisinone, Treatment, Long-term outcomes, Newborn screening

## Abstract

**Background:**

Tyrosinemia type 1 (TYR1) is a rare autosomal recessive disorder of amino acid metabolism that is fatal without treatment. With medication (nitisinone) and dietary restrictions outcomes are improved. We conducted a systematic review to investigate if treatment with nitisinone following screening provides better long-term outcomes than treatment with nitisinone following symptomatic detection.

**Methods:**

We searched Web of Science, Medline, Pre-Medline, and Embase up to 23rd September 2016 for journal articles comparing clinical outcomes of TYR1 patients receiving earlier versus later nitisinone treatment. Two reviewers independently screened titles and abstracts, assessed full texts, and appraised study quality. Data extraction was performed by a single reviewer and checked by a second.

**Results:**

We included seven articles out of 470 unique records identified by our search. The seven articles included four studies (three cohort studies and one cross-sectional study). Study sample sizes ranged from 17 to 148. There is consistent evidence that nitisinone is an effective treatment for TYR1, and some evidence that earlier treatment with nitisinone and dietary restrictions within the first one or 2 months of life is associated with reduced need for liver transplantation, lower rates of renal dysfunction, fewer neurological crises, and fewer, shorter hospital admissions compared to later treatment. However, study quality was moderate to weak, with high risk of confounding and applicability concerns to the screening context. We conducted post hoc analyses to address these issues. Results suggested an association between earlier treatment and fewer liver transplants (earlier treatment: 0% of 10–24 patients; later treatment: 25–60% of 4–15 patients), but no impact on neurological crises. We found no effect of treatment timing on mortality in either the primary or post hoc analyses. Post hoc analyses of other health-related outcomes were not possible because of sample size or reporting.

**Conclusions:**

There is some evidence from observational studies that earlier treatment with nitisinone might be beneficial but this is subject to bias. The applicability of our findings to the screening context or clinical practice is limited as not all early-treated patients were identified by screening and late-treated groups included patients born prior to the availability of nitisinone.

**Electronic supplementary material:**

The online version of this article (doi:10.1186/s13023-017-0696-z) contains supplementary material, which is available to authorized users.

## Background

Tyrosinemia type 1 (TYR1, OMIM 276700) is a rare autosomal recessive disorder of amino acid metabolism that is caused by a defect in the final enzyme of the pathway of tyrosine degradation, namely fumarylacetoacetate hydrolase (FAH) [1]. About one person in 100,000 is affected with TYR1 globally, but incidence is more common in some regions, notably in Québec [2]. Ninety-five mutations have been described so far in TYR1 with different geographical and ethnic distributions [3]. Deficiency of FAH causes an accumulation of tyrosine and toxic metabolites succinylacetone (SUAC), maleylacetoacetate and fumarylacetoacetate [4]. TYR1 mainly affects the liver, kidneys and peripheral nerves [1]. Two extreme clinical phenotypes have been described: an “acute” form with symptoms starting during the first few months of life and early severe liver failure, and a “chronic” form resulting in more gradual liver disease and increased risk for hepatocellular carcinoma [2]. Clinical presentations can be variable, even in cases with the same underlying mutation [5]. The age at onset of symptoms broadly correlates with severity [6]. Without treatment, death from liver failure and recurrent bleeding, neurological crisis or hepatocellular carcinoma frequently occurs before the age of 10 years [7].

Until the early 1990s, the only strategies available to manage the symptoms of TYR1 were protein-restricted diets (usually low in phenylalanine, methionine and tyrosine) and liver transplantation. The efficacy of these strategies is variable. For example, 1-year survival rates of 51% have been reported for children placed on a restricted diet before 6 months of age [7], and 90% for those undergoing liver transplantation [8]. In 1992, an alternative treatment, nitisinone, was introduced [9]. Nitisinone inhibits 4-hydroxyphenylpyruvate dioxygenase, an enzyme that is upstream of FAH in the tyrosine degradation pathway, and reduces the formation of toxic metabolites. Studies have suggested that the treatment can have dramatic benefits, including normalisation of renal dysfunction, control of liver failure in up to 90% individuals, the reduction or even elimination of neurological crises, and survival into adulthood [10–13].

An apparent benefit of earlier versus later treatment has been reported in a number of cohort studies (i.e. from Québec [10] and the UK [14]). To justify the introduction of newborn blood spot (NBS) screening programmes for TYR1, there should be evidence that, next to a simple, safe, precise and validated screening test, treatment at a pre-symptomatic phase leads to better outcomes for the screened individual compared to treatment initiation following symptomatic detection. We have recently published a linked review regarding the current state of evidence relating to the test accuracy of newborn screening for TYR1 using SUAC as primary marker that highlights important limitations in the literature [15]. To date, no study has synthesized and quality appraised all of the available evidence regarding early and late treatment of TYR1 with nitisinone. Therefore, the aim of this review was to compare the outcomes of TYR1 patients with early (pre-symptomatic) nitisinone treatment to later (following symptomatic detection) nitisinone treatment in a systematic review to assess the question of early treatment benefit from a screening perspective.

## Methods

The protocol is registered at the PROSPERO International Prospective Register of Systematic Reviews (CRD42015026912).

### Search strategy

Systematic literature searches were undertaken in Web of Science (Core Collection), Medline (Ovid), Medline In-Process & Other Non-Indexed Citations (Ovid), and Embase (Ovid). We searched for terms relating to tyrosinemia and nitisinone (the full electronic search strategy can be found in Additional file 1). We also examined the reference lists of included studies and of previous reviews. Individuals and organisations were contacted for studies that were not in the public domain. The search was conducted on 14th September 2015 and updated on 23rd September 2016. No date limits were applied for the original search.

### Eligibility

English language journal articles which investigated people who have TYR1 comparing early (following screening) versus late (following presentation with symptoms) nitisinone treatment were included. Diagnosis of TYR1 in the early treated patients was following universal newborn screening, cascade testing or incidental detection during newborn screening for phenylketonuria (PKU). All outcomes of the treatment were recorded. Exclusion criteria were non-human studies, papers not available in English language, and letters, editorials and communications, grey literature or conference abstracts.

### Study selection and data extraction

Screening of titles and abstracts and full text assessment was conducted independently by two reviewers. Data extraction was performed by a single reviewer, and all data extraction forms were checked by a second reviewer. Disagreements at each stage were resolved by consensus, with the involvement of third reviewer when required.

### Quality appraisal

Quality appraisal was undertaken independently by two reviewers using the Effective Public Health Practice Project (EPHPP) quality assessment tool for quantitative studies [16] (see Additional file 1). The tool covers six domains: selection bias, study design, confounders, blinding, data collection methods, withdrawals & dropouts. Each domain is rated as weak, moderate, and strong quality. A global score is determined on the basis of these ratings: weak (two or more weak domains), moderate (one weak domain), strong (no weak domains). Disagreements were resolved by consensus, with the involvement of third reviewer when required.

### Data summary and synthesis

A narrative synthesis of study characteristics and outcomes in terms of mortality, liver disease and transplantation, renal dysfunction and rickets, hospital admissions and neurocognitive outcomes is provided for all included studies.

To address concerns about the applicability of included studies to the screening perspective as well as possible confounding factors, three post-hoc comparisons were explored. The aim was to a) summarise the findings which are most applicable to the question of the benefit of screening using comparison 1 below (excluding patients who did not receive nitisinone immediately after diagnosis), and b) to summarise comparisons corrected for the bias of systematic variation in disease severity by excluding cases in comparisons 2 and 3 below. The following three post hoc analyses considered different subsets of the available individual patient data of the included studies:

1. Nitisinone treatment immediately following diagnosis in screen-detected versus symptomatically presenting cases. This is the comparison of most interest when considering what the benefits of implementing screening may be, but may be biased in favour of screening if screening detects milder spectrum of disease.
2. Nitisinone treatment immediately following diagnosis in early (<2 months) symptomatically presenting versus late (≥2 months) symptomatically presenting cases. Whilst less applicable to the screening question, this comparison is not biased in favour of early detection as the spectrum of disease in early presenting cases will be the same or more severe.
3. Direct (<1 month) nitisinone initiation versus delayed nitisinone initiation (within a time frame most TYR1 patients present symptomatically; 1–12 months) following screen detection. Whilst also less applicable to the screening question, this comparison is not biased in favour of early detection as the test detects the same spectrum of disease.

In the post hoc analyses, proportions between two groups were compared using the chi-square test; in cases of expected values smaller than five, a Fisher exact test was used.

## Results

### Searching, sifting, and sorting

**Fig. 1:**
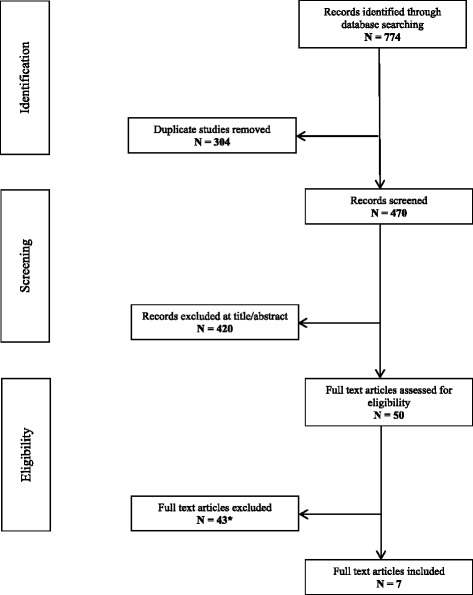
PRISMA flow diagram of records through the systematic review. *See Additional file 1 for list of excluded studies with reasons

Our electronic searches identified 470 unique records of which 50 full text articles were assessed (Fig. 1). Of these, 43 articles were excluded using the pre-defined inclusion/exclusion criteria (see Additional file 1 for excluded studies with reason). The remaining seven articles (reporting data from four studies) met the inclusion criteria and were included in the narrative synthesis.

### Characteristics of included studies

The main characteristics of studies included are summarised in Table 1. Three cohort studies (Birmingham study [12, 14, 17], Québec study [10, 11] and one international cohort [18]) and one cross-sectional study [19] published in seven papers reported on clinical outcomes in early versus late nitisinone-treated TYR1 patients. Data were collected retrospectively in the international cohort [18], in two papers from the Birmingham study [12, 14], and in one paper from the Québec study [11]. Data were collected prospectively in one paper from the Birmingham study [17], while both prospective and retrospective data were collected in the other paper from the Québec study [10] and the cross-sectional study [19]. The number of TYR1 patients included in the analyses ranged from 17 from a single centre in the UK [14] to 148 from 21 centres in Europe, Turkey and Israel [18]. There was a high overlap in included TYR1 cases between the two published papers from the Québec study [10, 11] with 78 of 95 patients (82%) included in both. TYR1 cases included in the three papers of the Birmingham (UK)-based study [12, 14, 17] overlapped widely. The TYR1 cases from Birmingham were possibly also included (at least in part) in the international cohort study [18] and in the multicentre, cross-sectional study [19]. Definition of ‘early’ and ‘late’ nitisinone treatment varied between the studies: in the Québec study, all early-treated patients were detected by universal newborn screening and nitisinone treatment was started before 30 days [10] or 4 weeks of age [11]. In the Birmingham (UK)-based study, the early treatment group mainly consisted of patients identified by cascade testing or coincidentally during routine PKU screening, with nitisinone treatment initiated before 2 months (10/11 screen-detected, one presenting with symptoms at the age of 1 month) [17], before 52 days (all screen-detected) [14] and before 2 weeks of age (all screen-detected) [12], respectively. The international cohort by Mayorandan et al. defined early treatment as nitisinone started before 1 month of age, but it was unclear if all patients were identified pre-symptomatically by screening [18]. In the multicentre, cross-sectional study by van Ginkel et al., the early treatment group consisted of pre-symptomatically diagnosed patients (cascade testing or routine PKU screening) with nitisinone started before 2 months of age [19]. Comparisons were made with TYR1 patients with ‘late’ nitisinone treatment initiation after 4 weeks or 30 days of age in the Québec study (21 of 26 patients were screen-detected but born prior nitisinone availability) [10, 11]. In the Birmingham study and the multicentre cross-sectional study, ‘late’ nitisinone treatment groups consisted of clinically presenting patients with nitisinone treatment started at 2–6 months or >6 months [17, 19], or between 1.5 and 17 months of age [14], whereas Santra et al. divided the symptomatically presenting cases by phenotype of liver disease at presentation (acute liver failure: nitisinone started between 1 month and 2 years; chronic liver disease: nitisinone started between 2 months and 9 years) [12]. Follow-up time in the cohort studies was on average 6.2 years and 13.6 years in the early and late intervention groups, respectively, in the analysis of the Québec cohort by Simoncelli et al. [11], and between five and 11 years in the early treatment group and between two and 19 years in the late treatment group in the analysis of the Québec cohort by Larochelle et al. [10] (data estimated by reviewers from bar chart only). Duration of follow-up was between one and 10 years in one part of the Birmingham study [12], between three and 19 years in surviving patients in another part of the Birmingham study [14], on average 9.1 years (standard deviation 6.3 years) in the international cohort [18] and not reported in a third paper of the Birmingham study [17].

**Table 1:** Characteristics of included studies

Study	Study design	Participants	Treatment
Birmingham cohort			
Bartlett 2014 [17]	Prospective cohort Follow-up time NR Study setting: Birmingham Children’s Hospital, UK Number of centres: 1	*N* = 38 TYR1 patients treated between 1989 and 2009. Pre-NTBC: *n* = 7 Post-NTBC: *n* = 31 Age at presentation: <2 months: *n* = 11 (6 detected by cascade testing, 4 incidental detection by routine PKU screening, 1 symptomatic presentation) 2–6 months: *n* = 11 >6 months: *n* = 9	Pre-NTBC: Diet Post-NTBC: NTBC and diet. NTBC: Initial dose of 1 mg/kg (0.6 mg/kg before 1995); dose adjusted to clinical and biochemical response (including plasma and urinary SUAC) and plasma levels thereafter (target 50 μmol/l). Diet NR
McKiernan 2015 [14]	Retrospective cohort (sibling-controlled) Age at last follow-up Pre-clinically diagnosed: 3–12.5 years Clinically diagnosed: 10–19 years or death at 1.5 and 7 months, respectively Study setting: Birmingham Children’s Hospital, UK Number of centres: 1	*N* = 17 TYR1 patients treated pre-symptomatically with NTBC following selective newborn screening and their clinically presenting siblings. Pre-clinically diagnosed: *n* = 12 NTBC start: median 4 (range 2–52) days. Clinically diagnosed siblings: *n* = 5 Age at presentation: Median 4 (range 1.5–17) months.	NTBC and diet NTBC: 1 mg/kg/day; NTBC titrated according to body weight until 10 kg, after which adjusted according to blood NTBC concentrations (3 monthly assessments). Diet: Fat soluble vitamin supplementation for at least 3 months. Tyr and Phe restriction. Breast feeding combined with Tyr and Phe free protein substitute. For formula fed infants, Tyr and Phe free protein substitute with natural protein requirements supplied as conventional formula. Dietary treatment titrated according to blood Phe and Tyr levels (3 monthly assessments).
Santra 2008 [12]	Retrospective cohort Follow-up: 1–10 years. Study setting: Birmingham Children’s Hospital, UK Number of centres: 1	*N* = 21 TYR1 patients treated with NTBC for at least 12 months. Phenotype of liver disease at presentation: Acute liver failure: *n* = 9 Age at presentation: Median 17 weeks (range 1 month to 2 years). Chronic liver disease: *n* = 7 Age at presentation: Median 60 weeks (range 2 months to 9 years). Pre-clinically: *n* = 5 Age at presentation: Median < 1 week (range < 1 to 2 weeks).	NTBC and diet NTBC: Standard protocol using nitisinone dosing of 0.6 mg/kg before 1995 and 1 mg/kg since 1995, the dose thereafter adjusted according to response. Diet: Normocaloric Tyr- and Phe-restricted diet, fat-soluble vitamins in the presence of liver dysfunction and phosphate supplements during hypophosphataemia.
Québec cohort			
Larochelle 2012 [10]	Cohort (Before 1994 retrospective, thereafter prospective data collection) Age at last follow-up (2009, death or OLT) No NTBC: OLT/death at 0.5–10 years; >30 days: 9–19 years or OLT/death at 2–8 years; ≤30 days: 5–11 years (estimated by reviewers from bar chart) Study setting: Québec, Canada Number of centres: NR	*N* = 78 TYR1 patients born 1984–2004. No NTBC: *n* = 28 (777 patient months) NTBC introduced ≤30 days: *n* = 24 (all detected by routine TYR1 screening) (2593 patient months). >30 days: *n* = 26 (21/26 detected by routine TYR1 screening) (535 patient months pre-NTBC; 3138 patient months with NTBC).	No NTBC: Diet (see below) Early- and Late-NTBC: NTBC and diet NTBC: Initially fixed at 0.6 or 1.0 mg/kg daily in 2 daily oral doses. For the first 2 years of the study: recrystallized preparation of NTBC. Thereafter: commercially-produced nitisinone. After 1999: NTBC dose titrated in order to minimise urine SUAC levels. Diet: Dietary restriction of Phe and Tyr aiming to maintain plasma Tyr at 200–400 μmol/L.
Simoncelli 2015 [11] (supersedes Larochelle et al. [10])	Retrospective cohort Follow-up until death or 1 January 2009; Age at study end date (1 January 2009) No NTBC: IQR 16.3–21.7 years NTBC ≥4 weeks: IQR 12.6–15.0 years NTBC <4 weeks: IQR 3.4–8.5 years (*p* < 0.001, Kruskal-Wallis test) Study setting: Québec, Canada Number of centres: 5	*N* = 95 TYR1 patients treated between 1984 and 2008. No NTBC: *n* = 28 NTBC introduced <4 weeks: *n* = 41 Median 13 days (IQR 11–16 days) (all detected by routine TYR1 screening). ≥4 weeks: *n* = 26 Median 1.0 years (IQR 0.4–2.2 years) (21/26 detected by routine TYR1 screening)	No NTBC: Diet and “curative” OLT. Early- and Late-NTBC: NTBC and diet. (NR, possibly as in Larochelle et al. [7])
International studies			
Mayorandan 2014 [18]	Retrospective cohort (Retrospective data collected via questionnaire) Average follow-up time: 9.1 (SD 6.3) years. Study setting: Europe, Turkey and Israel. Number of centres: 21	*N* = 168 included in study. TYR1 patients with questionnaire data. No NTBC: *n* = 10 NTBC: *n* = 154 (1157 patient years) No data on treatment: *n* = 4 *N* = 148 NTBC-treated patients included in analysis: NTBC start: <1 month: *n* = 37 (unclear if detected following screening or symptomatically) 1–6 months: *n* = 45 7–12 months: *n* = 20 >12 months: *n* = 46	Varying NTBC and diet treatment strategies between different centres. NTBC (*n* = 154): Initial NTBC dosage: Mean 1.7 mg/kg/day (SD 0.5, range 0.2–5) Current maintenance therapy: Mean 1.0 mg/kg/day (SD 0.3, range 0.3–2) 1–3 doses per day, on average 2 doses per day. Diet: All patients received dietary treatment in addition to NTBC. Natural protein restriction or Phe and Tyr calculation in 38%, natural protein restriction or Tyr intake calculation in 19%, natural protein restriction only in 19%, and Tyr and Phe calculation in 24% of centres.
Van Ginkel 2016 [19]	Cross-sectional (retrospective and prospective data collection) Study setting: Netherlands, Belgium, UK Number of centres: NR (multicentre)	*N* = 38 included in study 19 TYR1 patients and 19 age- and gender matched healthy controls. Included in subgroup analysis: *N* = 17–19 TYR1 patients Age at diagnosis <2 months (pre-symptomatically): *n* = 8–10 (detected by cascade testing or as coincidence in routine PKU screening) 2–6 months: *n* = 6 >6 months: *n* = 3	NTBC and diet Varying NTBC and diet treatment strategies between different centres. Diet: Phe and Tyr restricted. Natural protein restriction. Supplementation of all non-Phe and non-Tyr amino acids provided with one of the available amino acid mixtures that contain neither Phe nor Tyr.

*IQR* interquartile range, *NR* not reported, *NTBC* 2-(2-nitro-4-trifluoromethylbenzoyl)-1,3-cyclohexanedione, nitisinone, *OLT*, orthotopic liver transplantation, *Phe* phenylalanine, *PKU* phenylketonuria, *SD* standard deviation, *SUAC* succinylacetone, *Tyr* tyrosine, *TYR1* Tyrosinemia type 1

### Quality appraisal

Using the EPHPP quality assessment tool for quantitative studies [16], the overall methodological quality was judged as moderate (one weak rating) in the Québec study [10, 11], moderate to weak (one and three weak ratings, respectively) in the Birmingham study [12, 14, 17], and weak (three weak ratings each) in the international cohort [18] and the cross-sectional study [19] (see Table 2). There was high risk of selection bias in the cross-sectional study [19], the international cohort [18] and in one paper from the Birmingham study [14]. Dose of nitisinone and dietary treatment varied within the multicentre studies [18, 19] and between the studies. All four studies had a high potential of confounding as important factors (i.e. pre-existing health problems, presenting form of TYR1, compliance with treatment, co-treatment) were not controlled in study design or analysis. Length of follow-up differed between and within studies with usually higher age at last follow-up of (surviving) late-treated patients in the Birmingham [14, 17] and Québec [10, 11] cohort studies.

**Table 2:** Study quality of included studies according to EPHPP quality assessment tool [16]

Study	Global rating from sections A-F						Global rating for this study
	A) Selection bias	B) Study design	C) Confounders	D) Blinding	E) Data collection methods	F) Withdrawals and drop-outs	
Québec study							
Larochelle 2012 [10]	Strong	Moderate	Weak	Moderate	Strong	Strong	Moderate
Simoncelli 2015 [11]	Strong	Moderate	Weak	Moderate	Strong	Strong	Moderate
Birmingham study							
Bartlett 2014 [17]	Strong	Moderate	Weak	Moderate	Strong	Strong	Moderate
McKiernan 2015 [14]	Weak	Moderate	Weak	Moderate	Weak	Strong	Weak
Santra 2008 [12]	Moderate	Moderate	Weak	Moderate	Weak	Weak	Weak
International cohort							
Mayorandan 2014 [18]	Weak	Moderate	Weak	Moderate	Weak	Strong	Weak
Cross-sectional study							
Van Ginkel 2016 [19]	Weak	Weak	Weak	Moderate	Strong	Strong	Weak

The applicability of the reported findings to the screening context in our review question and to actual clinical practice is limited: none of the four included studies provided a comparison of outcomes of nitisinone treatment following TYR1 detection by universal newborn screening versus immediate nitisinone initiation following symptomatic presentation in an unscreened population. In the Québec study [10, 11], all of the patients with TYR1 in the early treatment group were identified by universal newborn screening but patients with TYR1 in the late treatment group (after 1 month of age, *n* = 26) included 21 children (81%) who were also screen-detected but diagnosed before nitisinone became available and treated with diet and supportive treatment only for up to 7 years. Only five patients in this group presented clinically with symptoms and were treated with nitisinone as soon as diagnosis was confirmed. In the UK-based cohort and the cross-sectional study, all [12, 19], 10/11 [17], and 11/12 [14] TYR1 patients in the early-treated group, respectively, were identified by cascade testing or during neonatal PKU screening and compared to patients who presented clinically. One in five [14] and 1 in 9 [19] patients included in the late-treated group, respectively, were diagnosed before nitisinone became available. In the international cohort study by Mayorandan et al. [18], it was unclear if all patients in the early-treated group were identified pre-symptomatically by newborn screening and if late-treated patients were treated with nitisinone directly after diagnosis.

### Mortality rate

Four articles reported on comparisons of the mortality rate between early and late nitisinone -treated TYR1 patients from the Québec cohort [10, 11], the Birmingham cohort [14], and the international cohort [18]. Combined, a total of 260 TYR1 patients (17 from the Birmingham cohort, 95 from the Québec cohort, and 148 from the international cohort) were included in the analyses. In the Québec study, ten people (36%) who never received nitisinone died at a median age of 1.1 years (interquartile range [IQR] 0.6–2.1 years), two deaths (8%) due to complications of liver transplantation occurred in the late nitisinone-treated group at ages of 7.4 and 8.7 years; none of the 24 people in the early nitisinone-treated group had died after a follow-up time of 5–11 years [10, 11]. There were significantly more deaths in the never nitisinone-treated group than either the early or late nitisinone-treated groups. Contrary to Larochelle and colleagues, our analysis indicated no significant difference in death rates between the early and late nitisinone-treated groups (*p* = 0.49 using Fisher exact test). In the Birmingham cohort, no death (0/12) occurred in the pre-symptomatically treated group after a follow-up time of 3–12.5 years, while two of five clinically presenting infants died. (One was born prior to nitisinone availability and died prior to liver transplantation aged 1.5 months, the other was born at 25 weeks’ gestation and died from respiratory complications of prematurity aged 7 months.) The age at last follow-up of the three surviving patients was 10–19 years [14]. In the international cohort, odds ratios (OR) for death were not significantly different in patients diagnosed and treated with nitisinone beyond the perinatal period compared to those with treatment initiation before the age of 1 month [18]. Age of patients at last follow-up or length of follow-up was not reported by comparison group. Only three deaths occurred among all 148 nitisinone-treated TYR1 patients (one patient was diagnosed and treated at the age of 5 years but died from failure of the transplanted liver, and two patients with treatment initiation between 7 and 12 months of age died from hepatocellular carcinoma and metastasis).

### Hepatic manifestations of TYR1 and requirement for liver transplantation

Five articles reported on comparisons in hepatic manifestations of TYR1 and/or the requirement for orthotopic liver transplantation (OLT) between early and late nitisinone-treated patients from the Québec study [10, 11], the Birmingham study [14, 17] and the international cohort [18] (see Table 3). The number of included patients ranged from 17 [14] to 148 [18]. In the Québec study, OLT was performed in 20 patients (71%) in the never nitisinone-treated group at a median age of 2.0 years (IQR 1.0–2.8 years), and in seven (27%) late nitisinone-treated patients at a median age of 4.8 years (IQR 2.9–7.6 years); no early nitisinone-treated patient required OLT after 5–11 years of follow-up. Rates of OLT were significantly lower amongst early and late nitisinone-treated patients compared to those who were never treated with nitisinone, and lower in those receiving early nitisinone treatment than late nitisinone treatment [10, 11]. In the Birmingham study, six of the seven (86%) never nitisinone-treated patients required OLT at a median age of 61 months (range 19–126 months) while only seven of the 31 (23%) nitisinone-treated patients proceeded to OLT at a median age of 53 months (range 5–163 months) (*p* = 0.004) [17]. No patient (0/11) treated before 2 months of age, 3/11 (27%) patients presenting between 2 and 6 months and 4/9 (44%) patients presenting after 6 months of age subsequently underwent OLT. The patient age at study end date was estimated between 4.7–12.1 years, 7.8–21.2 years, and 9.0–23.2 years for the <2 months, 2–6 months and >6 months comparison groups, respectively. Another analysis of the Birmingham cohort by McKiernan et al. found that no patient in the pre-symptomatically treated group (*n* = 12) required OLT and all were reported as currently clinically normal with no clinical, biochemical or radiological evidence of liver disease aged 3–12.5 years at last follow-up [14]. In the five clinically presenting siblings, one failed to respond to nitisinone and underwent OLT at 5 months and repeat OLT aged 15 for chronic rejection while the other two surviving patients were clinically stable with compensated liver disease aged 10 and 17. The international cohort study demonstrated significantly lower rates of hepatocellular carcinoma, and requirements for OLT in TYR1 patients with nitisinone started before 1 month of age versus nitisinone started after 12 months. The study also demonstrated lower rates of acute liver disease and liver cirrhosis for those treated early (nitisinone started before 1 month) versus nitisinone started at 7–12 months. Hepatomegaly rates were also lower for those treated before 1 month versus all late nitisinone-treated groups [18].

**Table 3:** Liver disease and need for liver transplantation in early and late nitisinone-treated TYR1 patients

Study	Study design	Participants	Outcome		Age at last follow-up / study end date
Birmingham study					
Bartlett 2014 [17]	Cohort (prospective data collection)	N=38 No NTBC: n=7 NTBC: n=31 Age at presentation: <2 months: n=11 2-6 months: n=11 >6 months: n=9	*OLT*		
			<2 months:	0/11	NR
			2-6 months:	3/11 (27%)	NR
			>6 months:	4/9 (44%)	NR
			No NTBC:	6/7 (86%)	NR
				(p=0.004 vs 7/31 with NTBC)	
			Median age at NTBC start No OLT: 52 (range 2-990) days; OLT: 428 (range 86-821) days. (p=0.004)		
McKiernan 2015 [14]	Cohort (sibling-controlled, retrospective data collection)	N=17 Pre-clinically diagnosed: n=12 Clinically diagnosed siblings: n=5	*OLT*		
			Pre-clinically:	0/12	3-12.5 years;
			Clinically:	1/5 (20%)	10-19 years or death at 1.5 and 7 months, respectively
			*Liver disease*		
			Pre-clinically	0/12	3-12.5 years;
			Clinically	2/3 (67%) surviving patients	10-19 years
Québec study					
Larochelle 2012 [10]	Cohort (Before 1994 retrospective, thereafter prospective data collection)	N=78 NTBC introduced ≤30 days: n=24 >30 days: n=26 No NTBC: n=28	*UI*		
			≤30 days:	0/24	5-11 years;
			>30 days:	7/26 (27%)	9-19 years or OLT/death at 2-8 years;
			No NTBC:	20/28 (71%)	OLT/death at 0.5-10 years
			p < 0.001, ≤30 days vs No NTBC (Chi square test); p < 0.001, >30 days vs No NTBC (Chi square test); p = 0.010, ≤30 vs >30 days (Fisher exact test)^a^		
Simoncelli 2015 [11]	Cohort (Retrospective data collection)	N=95 <4 weeks: n=41 ≥4 weeks: n=26 No NTBC: n=28 Supersedes Larochelle et al. [10]	*OLT*		
			<4 weeks:	0/41	IQR 3.4-8.5 years
			≥4 weeks:	7/26 (27%)	IQR 12.6-15.0 years
			No NTBC:	20/28 (71%)	IQR 16.3-21.7 years
			(p<0.001, Fisher exact test)		(p<0.001, Kruskal-Wallis test)
International cohort					
Mayorandan 2014 [18]	International cohort (retrospective data collection via questionnaire)	N=168 included in study. N=148 NTBC-treated patients included in analysis. NTBC start: <1 month: n=37 1-6 months: n=45 7-12 months: n=20 >12 months: n=46	*OLT*	OR (95%CI)	
			>12 months:	12.7 (1.5-103)^b^	NR
			*Acute liver disease*		
			<1 month:	0/37	NR
			7-12 months:	3/20 (15%)*	NR
			*Liver cancer*	OR (95%CI)	
			>12 months:	12.7 (1.5-103)^b^	NR
			*Liver cirrhosis*	OR (95%CI)	
			7-12 months:	41.6 (2.2-779.9)^b^	NR
			>12 months:	40.5 (2.3-704.1)^b^	NR
			*Hepatomegaly*	OR (95%CI)	
			1-6 months:	3.3 (0.9-11.3)^b^	NR
			7-12 months:	4.4 (1.1-17.7)^b^	NR
			>12 months:	3.9 (1.1-13.3)^b^	NR
			Odds/rates for not presented late-treated groups were not significantly different compared to early NTBC (<1 month).		

^a^Based on our analysis of data presented in the paper by Larochelle et al. [10] using Fisher exact test. ^b^ <1 month: OR = 1. * *p* <0.05 vs <1 month

*CI* confidence interval, *IQR* interquartile range, *NR* not reported, *NTBC* nitisinone, *OLT* orthotopic liver transplantation, *OR* odds ratio

### Renal dysfunction and rickets

Two articles reported on renal dysfunction or rickets in early- and late-treated TYR1 patients [12, 18]. There were 148 patients included in the international cohort and 21 in the Birmingham cohort. Santra et al. reported that all TYR1 patients had proteinuria at presentation with high values even in children who were diagnosed pre-symptomatically [12]. Children presenting with acute liver failure were more likely to be hypophosphataemic (*p* < 0.01) and have excessive phosphaturia (*p* = 0.05) than children who were diagnosed asymptomatically, while children presenting with chronic hepatic and/or renal dysfunction had intermediate values. All three measured markers of tubular dysfunction normalised within 1 year of nitisinone treatment and remained normal at follow-up of up to 10 years. No child redeveloped tubular dysfunction after starting on nitisinone treatment.

The international cohort study found higher rates of renal dysfunction in TYR1 patients treated with nitisinone after 12 months of age (~24%, estimated from bar chart) compared to treatment initiation before 1 month of age (~7%, estimated from bar chart) (*p* < 0.05; OR 5.5; 95% confidence interval [CI] 1.1–26.6) [18]. Rickets were more frequently observed in TYR1 patients with nitisinone initiated after 12 months of age (~20%, estimated from bar chart) compared to early treated patients (no cases; *p* < 0.05; OR 19, 95% CI 1.1–338.3).

### Hospital admissions

Two articles reported on the effect of early and late nitisinone treatment on hospitalisations in 78 and 95 TYR1 patients from Québec [10, 11]. Early nitisinone treatment was associated with significant fewer hospital admissions, in terms of both number of admissions per person-year (0.83, 0.41, and 0.16 for never-nitisinone, late-nitisinone, and early-nitisinone groups, respectively; *p* < 0.001) and length of stay per person-year (7.6, 3.2, and 0.4 days, respectively; *p* < 0.001) [11]. The proportion of patients with neurological crises was significantly different between the three groups (14 [50%], 5 [19%], and 0 for never-nitisinone, late-nitisinone, and early-nitisinone groups, respectively; *p* < 0.001) [11]. No new acute hepatic or neurologic events occurred after the first dose of nitisinone, even in late-treated patients [10].

### Neurocognitive outcomes

Three articles reported on neurocognitive outcomes in early and late nitisinone-treated patients; two cohort studies [14, 18] and one cross-sectional study [19]. Seventeen patients were included in this analysis from the Birmingham study [14], 17 to 19 in the cross-sectional study [19] and 148 in the international cohort [18]. In the Birmingham cohort, some degree of learning difficulty was observed in 4/9 pre-symptomatically treated patients of school age [14]. All three surviving siblings who were symptomatically treated required extra educational support with one attending a specialised secondary school due to learning difficulties. Authors of the multicentre, cross-sectional study did not identify any significant differences in reaction time or percentage of errors in any of the neuropsychological tasks (intelligence quotient [IQ], executive functioning, social cognition) when comparing TYR1 patients based on the age at diagnosis (<2 months, *n* = 10; 2–6 months, *n* = 6; >6 months, *n* = 3) or pre-symptomatically (*n* = 10) versus symptomatically (*n* = 9) diagnosed patients [19]. While controlling for age, the duration of nitisinone treatment was inversely correlated with IQ (*r* = −0.51, *p* = 0.046) [19]. The international cohort [18] observed impaired psychomotor development in 30/148 (20%), hyperactivity/attention deficit syndrome or behavioural disorders in 12/148 (8%) and learning/language difficulties or dyslexia in 2/148 (1.4%) of all nitisinone-treated TYR1 patients. None of which were associated with time at which nitisinone was initiated.

### Post hoc comparisons

#### 1) screen detected vs symptomatically detected patients, direct nitisinone start after diagnosis

From the Québec study [10], we excluded 21 of 26 late-treated patients as they were born prior to nitisinone availability. This post hoc analysis considered 29 patients with immediate nitisinone treatment following (1) detection through screening (*n* = 24) and (2) symptomatic detection (*n* = 5). No benefit of early nitisinone treatment on mortality was observed in this post hoc analysis (no deaths occurred in either the 24 screen-detected or five symptomatically presenting cases). After the exclusion of 26 TYR1 patients born prior to the nitisinone era, benefits were observed of early nitisinone-treatment on the need for OLT (0/24 screened vs 3/5 symptomatically presenting patients needed OLT) (see Table 4 for a summary of initial and post hoc analyses results). While the proportion of patients with neurological crises differed significantly between the three groups in the initial analysis (*p* < 0.001, Fisher exact test [11]), the post hoc analysis did not demonstrate a difference in the number of patients with neurological crises between screen-detected and symptomatically presenting patients with immediate nitisinone treatment after diagnosis (no neurological crisis occurred in either groups). No post hoc analysis was possible for number of hospital admissions or days in hospital as individual patient data were not available.

**Table 4:** Summary of initial study results and post hoc analyses

	Death	Need for OLT	Patients with neurological crisis
*Initial analysis* Early vs late	*Québec cohort* Larochelle 2012 [10] 0/24 vs 2/26 (*p* = 0.49)^a^ *Birmingham study* McKiernan 2015 [14] 0/12 vs 2/5 *(p = 0.07)*	*Québec cohort* Larochelle 2012 [10] 0/24 vs 7/26 (*p* = 0.01)^a^ *Birmingham study* McKiernan 2015 [14] 0/12 vs 1/5 *(p = 0.29)* Bartlett 2014 [17] 0/11 vs 7/20 *(p = 0.033)*	*Québec cohort* Larochelle 2012 [10] 0/24 vs 5/26 *(p = 0.051)* Simoncelli 2015 [11] 0/41 vs 5/26 *(p = 0.007)*
*Post hoc 1* Screen detection vs symptomatic detection, all with direct nitisinone initiation	*Québec cohort* Larochelle 2012 [10] 0/24 vs 0/5 *Birmingham study* McKiernan 2015 [14] 0/12 vs 1/4 *(p = 0.25)*	*Québec cohort* Larochelle 2012 [10] 0/24 vs 3/5 *(p = 0.0027)* *Birmingham study* McKiernan 2015 [14] 0/12 vs 1/4 *(p = 0.25)* Bartlett 2014 [17] 0/10 vs 5/15 *(p = 0.061)*	*Québec cohort* Larochelle 2012 [10] 0/24 vs 0/5 Simoncelli 2012 [11] NA
*Post hoc 2* Early symptomatic vs late symptomatic,all with direct nitisinone initiation	*Québec cohort* Larochelle 2012 [10] NA *Birmingham cohort* NA	*Québec cohort* Larochelle 2012 [10] NA *Birmingham cohort* McKiernan 2015 [14] NA Bartlett 2014 [17] 0/1 vs 5/14	*Québec cohort* NA
*Post hoc 3* Screen detection, direct nitisinone initiation vs screen detection, 1–12 months delayed nitisinone initiation	*Québec cohort* Larochelle 2012 [10] 0/24 vs 0/10 *Birmingham cohort* NA	*Québec cohort* Larochelle 2012 [10] 0/24 vs 0/10 *Birmingham cohort* McKiernan 2015 [14] NA Bartlett 2014 [17] NA	*Québec cohort* Larochelle 2012 [10] 0/24 vs 1/10 *(p = 0.294)* Simoncelli 2015 [11] NA

^a^Based on our analysis of data presented in the paper by Larochelle et al. [10] using Fisher exact test

*NA* not applicable, *OLT* Orthotopic liver transplantation

The Birmingham cohort included small numbers for this post hoc analysis, which are reported in Table 4. No post hoc analysis was possible for the data presented by Santra et al. [12], for the international cohort by Mayorandan et al. [18] or the multicentre, cross-sectional study [19], as individual patient data on diagnostic procedures, delay in nitisinone initiation and/or clinical outcomes were not presented in these papers.

#### 2) early symptomatic vs late symptomatic detection, direct nitisinone start after diagnosis

This analysis was only possible using the Birmingham study [17], and included small numbers. We excluded 10 screen-detected patients as well as six patients presenting symptomatically prior to the availability of nitisinone (*n* = 3) or with delayed nitisinone treatment for other (unknown) reasons (*n* = 3). This post hoc comparison therefore considered one case presenting early with symptoms at the age of 1 month and 14 patients presenting with symptoms after 2 months of age, all were treated with nitisinone immediately after diagnosis. The patient presenting early with symptoms did not require OLT after follow-up of 7.5 years, while 5/14 (36%) of the patients presenting later (≥2 months) with symptoms proceeded to OLT at a median age of 3.3 years (range 0.4–13.6 years).

#### 3) direct vs 1–12 months delayed nitisinone initiation in screen-detected patients

For this comparison, we used all screen-detected patients from the Québec study [10] who started nitisinone within the first month of life (*n* = 24) or within a time frame that most patients present symptomatically (1–12 months, *n* = 10). We excluded 16/26 patients from the late-treated group as they were not screen-detected (*n* = 5) or nitisinone was initiated after 12 months of age (*n* = 11). No deaths occurred in either of the direct or delayed treatment groups, and no patients in the either group needed OLT. None of the patients directly treated with nitisinone had a neurological crisis, 1/10 (10%) of the patients with delayed nitisinone-treatment had a neurological crisis, which occurred before nitisinone initiation.

## Discussion

We examined the clinical outcomes for people with TYR1 who received early treatment with nitisinone (when they were pre-symptomatic) versus late treatment (after presenting with symptoms). Four studies (published in seven papers; three cohort studies and one cross-sectional study) provided data on clinical outcomes. Results from Canada [10, 11], England [14, 17] and an international cohort study [18] suggest an association between earlier nitisinone treatment and lower rates of liver-related sequelae and liver transplantation, but no effect on mortality. However, all of the comparisons are subject to significant bias. Our post-hoc analysis to control for some of the confounding where possible could not replicate the same effects. The effects of early versus later nitisinone treatment on cognitive outcomes were inconsistent across studies. No significant differences in cognition were found between treatment groups in the international cohort [18] and the multicentre, cross-sectional study [19], and no benefits of early treatment with nitisinone was reported for learning difficulties in a small subgroup of the Birmingham cohort [14] (no statistical test was performed). An observed negative correlation between duration of nitisinone treatment and IQ [19] might indicate a possible detrimental effect on cognitive outcomes.

A clear understanding of the potential benefits of earlier treatment with nitisinone is difficult to ascertain because of limitations in the evidence base. First, all of the studies we identified were of observational design. There are numerous methodological issues associated with observational studies including selection bias, lack of control of participant characteristics, and absence of comparator treatments. Second, sample sizes were often small and participant populations heterogeneous, which limit the generalizability of results. Third, there are concerns regarding the applicability of the findings to the screening context in our review question and to actual clinical practice as none of the four included studies provided a comparison of clinical outcomes of early nitisinone treatment following universal newborn screening using SUAC as primary marker versus immediate treatment with nitisinone following symptomatic presentation in unscreened children. These types of limitations are inherent in research into rare diseases, and it is important to bear in mind that TYR1 is a very rare disease (the total number of TYR1 patients treated with nitisinone in Europe is fewer than 400 [18]). This raises important questions about research with people who have rare diseases. For example, what level of evidence do we require, and how do we deal with bias in small observational studies? Gagne and colleagues [20] have reviewed methods for generating evidence on health outcomes for people who have rare diseases, and identified two broad approaches to meeting these challenges. The first approach focusses on study designs that keep the total number of participants to a minimum. The second approach is to employ designs that maximise the number of participants who receive the study treatment, such as crossover and N-of-1 trials (which enable all participants to receive treatment). Bogaerts and colleagues [21] have also suggested a range of alternatives, including an increased tolerance for type 1 errors, and employing Bayesian designs (focusing on estimation rather than hypothesis testing).

Our systematic review has a number of strengths. We conducted a wide ranging and exhaustive systematic searches without date limit, obtained expert input, and two reviewers conducting all processes (literature screening, data extraction and quality assessment). However, there are also some limitations. First, we excluded articles not available in the English language; non-English-language papers may be available and add further information. Second, the exclusion criteria of our post hoc analyses were not pre-specified in the protocol and the results should be interpreted with caution. Third, the information used in our post hoc analysis was estimated for two studies; delay of nitisinone treatment from a bar chart (see figure two in Larochelle et al. [10]), and a table (see table two in Bartlett et al. [17]).

A previous rapid review of screening for TYR1 concluded that nitisinone is an effective treatment and that it leads to better outcomes when initiated early [22]. At the time of this review, the only published literature on earlier versus later treatment with nitisinone was the Canadian cohort study of Larochelle et al. [10]. The results of our full systematic review, which included an additional three studies, are broadly consistent with this. However, we have identified issues with the evidence base that temper any conclusions about whether earlier treatment is more beneficial than later treatment.

To our knowledge, this is the first systematic review that provides a synthesis of all of the available studies that have compared the outcomes of TYR1 patients with early (pre-symptomatic) nitisinone treatment to later nitisinone-treated (symptomatically presenting) patients to assess the question of early treatment benefit from a screening perspective. Adequate evidence is still very limited. Further investigation is needed regarding whether the TYR1 cases detected by screening represent the same spectrum of disease as those detected symptomatically and whether it is certain that all screen-detected babies would become symptomatic in the absence of screening. Evidence is needed on whether improved outcomes with early administration of nitisinone are due to the effectiveness of the drug, differences in the spectrum of disease or other confounding factors. Observational research is very valuable for rare diseases, particularly where international collaboration produces increased sample sizes, but efforts must be made to adjust for confounders if results are to be interpreted meaningfully.

## Conclusions

Nitisinone appears to be an effective treatment for TYR1. There is some evidence that liver transplants are less common amongst individuals who receive treatment during the first 2 months of life than those who receive it later. This is supported by post hoc comparisons attempting to control for the effect of confounders and addressing applicability issues. Further research is needed to strengthen the evidence base and confirm the benefit of pre-emptive nitisinone treatment.

## Additional file

Additional file 1: Table S1. Search strategy for Ovid Medline. This table shows the electronic search strategy developed for Medline (Ovid) and the number of hits retrieved per line. **Figure S1**. EPHPP quality assessment tool for quantitative studies. This file shows the EPHPP “Quality assessment tool for quantitative studies” that was used to appraise the quality of all included studies. **Table S2**. Excluded studies with reason. This table lists all studies that were excluded at full text stage and reasons for their exclusion. (DOCX 126 kb)

## Abbreviations

CI: Confidence interval
EPHPP: Effective public health practice project
FAH: Fumarylacetoacetate hydrolase
IQ: Intelligence quotient
IQR: Interquartile range (central 50% of values within the data set)
NA: Not applicable
NBS: Newborn blood spot
NR: Not reported
NTBC: Nitisinone
OLT: Orthotopic liver transplantation
OR: Odds ratio
PKU: Phenylketonuria
SUAC: Succinylacetone
TYR1: Tyrosinemia type 1
