# Supplementary material for: Evaluation of pre-symptomatic nitisinone treatment on long-term outcomes in Tyrosinemia type 1 patients: a systematic review
Source: Orphanet J Rare Dis. 2017 Sep 11;12:154. doi: 10.1186/s13023-017-0696-z (PMC5594482; doi:10.1186/s13023-017-0696-z)
Supplement: Additional file 1: Table S1. — Search strategy for Ovid Medline. This table shows the electronic search strategy developed for Medline (Ovid) and the number of hits retrieved per line. Figure S1. EPHPP quality assessment tool for quantitative studies. This file shows the EPHPP “Quality assessment tool for quantitative studies” that was used to appraise the quality of all included studies. Table S2. Excluded studies with reason. This table lists all studies that were excluded at full text stage and reasons for their exclusion. (DOCX 126 kb) [file 13023_2017_696_MOESM1_ESM.docx]

**Additional file**

**Search strategy for Ovid Medline**

Search strategies were developed for MEDLINE (Ovid) and were adapted appropriately for other databases: MEDLINE In-Process & Other Non-Indexed Citations (Ovid), EMBASE (Ovid), and Web of Science.

| **Searches** | **Results** | **Search Type** | **Actions** |
| --- | --- | --- | --- |
|  | 1 | exp Tyrosinemias/ | 309 |
|  | 2 | (tyrosinemia* or tyrosinaemia*).mp. [mp=title, abstract, original title, name of substance word, subject heading word, keyword heading word, protocol supplementary concept word, rare disease supplementary concept word, unique identifier] | 999 |
|  | 3 | (tyr1 or tyr-1).mp. [mp=title, abstract, original title, name of substance word, subject heading word, keyword heading word, protocol supplementary concept word, rare disease supplementary concept word, unique identifier] | 963 |
|  | 4 | (tyri or tyr-i).mp. [mp=title, abstract, original title, name of substance word, subject heading word, keyword heading word, protocol supplementary concept word, rare disease supplementary concept word, unique identifier] | 29 |
|  | 5 | (((fumarylacetoacetate adj hydrolase) or fumarylacetoacetase or fah) adj2 deficien*).mp. [mp=title, abstract, original title, name of substance word, subject heading word, keyword heading word, protocol supplementary concept word, rare disease supplementary concept word, unique identifier] | 127 |
|  | 6 | 1 or 2 or 3 or 4 or 5 | 2008 |
|  | 7 | Nitisinone.mp. | 144 |
|  | 8 | 104206 65 7 Nitisinone.rn. | 127 |
|  | 9 | ntbc.mp. | 120 |
|  | 10 | orfadin.mp. | 4 |
|  | 11 | 2 nitro 4 trifluoromethylbenzoyl.mp. | 45 |
|  | 12 | 7 or 8 or 9 or 10 or 11 | 181 |
| **** | **13** | **6 and 12** | **140** |

**Supplement 2** EPHPP quality assessment tool for quantitative studies

**Supplement 3** Excluded studies with reason (n = 43)

| **Reference** | **Reason for exclusion** |
| --- | --- |
| **Original search (n = 40)** | |
| 1. Alobaidy HA, Yahya NA, Said RM. Tyrosinemia type 1: Clinical and biochemical analysis of cases with poor treatment outcome. Jordan Medical Journal. 2011;45(2):205-12. | Case reports of 3 TYR1 cases |
| 1. Alvarez, F., et al. (2005). "NITISINONE (NTBC) TREATMENT OF HEPATORENAL TYROSINEMIA IN QUEBEC." Journal of Inherited Metabolic Disease **28**: 49-49. | Conference abstract |
| 1. Anonymous. Nitisinone. Type 1 tyrosinemia: An effective drug. Prescrire International. 2007;16(88):56-8. | Duplicate |
| 1. Anonymous. Nitisinone. Australian Prescriber. 2009;32(2):54-5. | No early vs late NTBC comparison, not a systematic review |
| 1. Anonymous. Nitisinone: new drug. Type 1 tyrosinemia: an effective drug. Prescrire International. 2007;16(88):56-8. | Not a systematic review |
| 1. Arora N, Stumper O, Wright J, Kelly DA, McKiernan PJ. Cardiomyopathy in tyrosinaemia type I is common but usually benign. Journal of Inherited Metabolic Disease. 2006;29(1):54-7. | No early vs late NTBC comparison |
| 1. Barkaoui, E., et al. (2010). "HEREDITARY TYROSINEMIA TYPE I: LIVER TRANSPLANTAION (LT) WITH LIVING DONOR FOR HEPATOCARCINOMA AFTER 7 YEARS OF NTBC." Pediatric Research **68**: 401-401. | Conference abstract |
| 1. Baumann U, Rodeck B. Liver transplantation in tyrosinaemia type I. Monatsschrift Kinderheilkunde. 2004;152(10):1102-6. | German language |
| 1. Bendadi, F., et al. (2014). "Impaired cognitive functioning in patients with tyrosinemia type i receiving nitisinone." Journal of Pediatrics 164(2): 398-401. | No early vs late NTBC comparison |
| 1. Buckley BM. Clinical trials of orphan medicines. The Lancet. 2008;371(9629):2051-5. | No early vs late NTBC comparison |
| 1. Choy, Y. S., et al. (2006). "Variable response of patients with hepatorenal tyrosinemia to NTBC." Journal of Inherited Metabolic Disease 29: 108-108. | Conference abstract |
| 1. Couce, M. L., et al. (2011). "Tyrosinemia type 1 in Spain: Mutational analysis, treatment and long-term outcome." Pediatrics International 53(6): 985-989. | No early vs late NTBC comparison |
| 1. De Laet C, Terrones Munoz V, Jaeken J, Francois B, Carton D, Sokal EM, et al. Neuropsychological outcome of NTBC-treated patients with tyrosinaemia type 1. Developmental Medicine and Child Neurology. 2011;53(10):962-4. | Letter, no early vs late NTBC comparison |
| 1. El-Karaksy, H. K., et al. (2008). "NTBC therapy for tyrosinemia type 1: How much is enough?" Journal of Hepatology 48: S332-S332. | Conference abstract |
| 1. Elpeleg ON, Shaag A, Holme E, Zughayar G, Ronen S, Fisher D, et al. Mutation analysis of the FAH gene in Israeli patients with tyrosinemia type I. Human mutation. 2002;19(1):80-1. | No early vs late NTBC comparison |
| 1. Gissen P, Preece MA, Willshaw HA, McKiernan PJ. Ophthalmic follow-up of patients with tyrosinaemia type I on NTBC. Journal of Inherited Metabolic Disease. 2003;26(1):13-6. | No early vs late NTBC comparison |
| 1. Hercbian, D., et al. (2009). "TYROSINEMIA TYPE 1: THERAPEUTIC DRUG MONITORING OF NTBC IN PLASMA/SERUM USING LIQUID CHROMATOGRAPHY COUPLED TO TANDEM MASS SPECTROMETRY." Molecular Genetics and Metabolism 98(1-2): 19-19. | Conference abstract |
| 1. Holme E, Lindstedt PS, Lock EA. Treatment of tyrosinemia type I with an enzyme inhibitor (NTBC). International Pediatrics. 1995;10(1):41-3. | No early vs late NTBC comparison |
| 1. Holme E, Lindstedt S. Tyrosinaemia type I and NTBC (2-(2-nitro-4-trifluoromethylbenzoyl)-1,3- cyclohexanedione). Journal of Inherited Metabolic Disease. 1998;21(5):507-17. | No early NTBC group following screening; only NTBC before/after 2 years of age |
| 1. Holme E, Lindstedt S. Nontransplant treatment of tyrosinemia. Clinics in Liver Disease. 2000;4(4):805-14. | Excluded as no early (screened) vs late NTBC data |
| 1. Joshi SN, Venugopalan P. Experience with NTBC therapy in hereditary tyrosinaemia type I: An alternative to liver transplantation. Annals of Tropical Paediatrics. 2004;24(3):259-65. | No early vs late NTBC comparison |
| 1. Kitagawa T. Hepatorenal tyrosinemia. Proceedings of the Japan Academy Series B: Physical and Biological Sciences. 2012;88(5):192-200. | No systematic review, no early vs late NTBC comparison |
| 1. Masurel-Paulet A, Poggi-Bach J, Rolland MO, Bernard O, Guffon N, Dobbelaere D, et al. NTBC treatment in tyrosinaemia type I: Long-term outcome in French patients. Journal of Inherited Metabolic Disease. 2008;31(1):81-7. | Early NTBC group (<6 months) is not screen-detected; comparison of acute vs sub-acute vs chronic forms of TYR1 |
| 1. McKiernan PJ, Preece MA, Green A, Lindstedt S, Holme E, Lock EA, et al. IMPROVEMENT IN LIVER-FUNCTION AND HISTOLOGY IN TYROSINEMIA TYPE-1 WITH NTBC. Hepatology. 1995;22(4):1076-. | Conference abstract |
| 1. McKiernan, P. J., et al. (1998). "Success of NTBC in infantile liver failure due to tyrosinaemia type 1." Hepatology 28(4): 422A-422A. | Conference abstract |
| 1. McKiernan PJ. Nitisinone in the treatment of hereditary tyrosinaemia type 1. Drugs. 2006;66(6):743-50. | No early vs late NTBC comparison |
| 1. McKiernan, P. J. (2013). "Nitisinone for the treatment of hereditary tyrosinemia type i." Expert Opinion on Orphan Drugs 1(6): 491-497. | Not a systematic review, no early vs late NTBC comparison |
| 1. Nakamura K, Matsumoto S, Mitsubuchi H, Endo F. Diagnosis and treatment of hereditary tyrosinemia in Japan. Pediatrics International. 2015;57(1):37-40. | No early vs late NTBC comparison |
| 1. Pierre G, Chronopoulou E. Metabolic disorders presenting as liver disease. Paediatrics and Child Health (United Kingdom). 2013;23(12):509-15. | No early vs late NTBC comparison |
| 1. Pohorecka, M., et al. (2008). "Pharmacokinetics of NTBC (nitisinone) following a single dose to 5 children with tyrosinemia type 1." Journal of Inherited Metabolic Disease 31: 5-5. | Conference abstract |
| 1. Pohorecka, M., et al. (2012). "Behavioral and intellectual functioning in patients with tyrosinemia type I." Endokrynologia, Diabetologia i Choroby Przemiany Materii Wieku Rozwojowego 18(3): 96-100. | Still to check! |
| 1. Van Spronsen FJ, Bijleveld CMA, Van Maldegem BT, Wijburg FA. Hepatocellular carcinoma in hereditary tyrosinemia type I despite 2-(2 nitro-4-3 trifluoro- methylbenzoyl)-1, 3-cyclohexanedione treatment. Journal of Pediatric Gastroenterology and Nutrition. 2005;40(1):90-3. | Case report; no early vs late NTBC comparison |
| 1. Raimann, E., et al. (2006). "Tyrosinemia type 1, follow up of 8 patients treated with phenylalanine (Phe) and tyrosine (Tyr) restricted diet and NTBC." Journal of Inherited Metabolic Disease 29: 108-108. | Conference abstract |
| 1. Santra, S. and U. Baumann (2008). "Experience of nitisinone for the pharmacological treatment of hereditary tyrosinaemia type 1." Expert Opinion on Pharmacotherapy 9(7): 1229-1236. | Not a systematic review; no early vs late NTBC comparison |
| 1. Scott, C. R., et al. (2000). "The effectiveness of 2-(2-nitro-4-trifluoromethylbenzoyl)-1,3-cyclohexanedione (NTBC) as a therapeutic agent for tyrosinemia-I: The US experience." Pediatric Research 47(4): 168A-168A. | Conference abstract |
| 1. Thimm, E., et al. (2011). "Increase of CSF tyrosine and impaired serotonin turnover in tyrosinemia type I." Molecular Genetics and Metabolism 102(2): 122-125. | 3 patients only; no early vs late NTBC comparison |
| 1. Thimm, E., et al. (2012). "Neurocognitive outcome in patients with hypertyrosinemia type i after long-term treatment with NTBC." Journal of Inherited Metabolic Disease 35(2): 263-268. | No early vs late NTBC comparison |
| 1. Van Spronsen, F. J., et al. (2005). "Hepatocellular carcinoma in hereditary tyrosinemia type I despite 2-(2 nitro-4-3 trifluoro- methylbenzoyl)-1, 3-cyclohexanedione treatment." Journal of Pediatric Gastroenterology and Nutrition 40(1): 90-93. | Case report |
| 1. Wijburg FA, Reitsma Ch WC, Slooff MJH, Van Spronsen FJ, Koetse HA, Reijngoud DJ, et al. Liver transplantation in tyrosinaemia type I: The Groningen experience. Journal of Inherited Metabolic Disease. 1995;18(2):115-8. | No early vs late NTBC comparison |
| 1. Wilson, C. J., et al. (2000). "Phenylalanine supplementation improves the phenylalanine profile in tyrosinaemia." Journal of Inherited Metabolic Disease 23(7): 677-683. | No early vs late comparison; no clinical outcomes |
| 1. Zaman, T. and R. Moradian (2006). "Long term follow up of eight patients affected of tyrosinemia type I enrolled in the international NTBC trials, treated with NTBC and tyrosine restricted diet." Journal of Inherited Metabolic Disease 29: 110-110. | Conference abstract |
| **Updated search (n = 3)** | |
| 1. Alobaidy, H. and E. Barkaoui (2015). "Experience of a Single Center in NTBC Use in Management of Hereditary Tyrosinemia Type I in Libya." Iranian Journal of Pediatrics 25(5): e3608. | No early vs late NTBC comparison |
| 1. Mannion, M., et al. (2016). "Type 1 tyrosinaemia." Irish Medical Journal **109**(5). | All 3 cases treated early (<2 weeks) |
| 1. Shah, I. and F. Shah (2016). "Tyrosinemia type I: Case series with response to treatment to NTBC." Indian Journal of Gastroenterology **35**(3): 229-231. | All 4 cases treated late |
